# Notes on Preparations for the Sick

**Published:** 1895-12

**Authors:** 


### Notes on Preparations for the Sick.

**Chlor-anodyne.**—PARKE, DAVIS & CO., London.—In consequence of the great variation in the composition of the various preparations which may be dispensed for Chlorodyne, an attempt to provide an efficient and elegant substitute of uniform composition has led to the adoption of the above name for Messrs. Parke, Davis & Co.'s compound. Each fluid ounce contains:

| | | | |
|---|---|---|---|
| Morphine Muriate ... | 2⅞ grains. | Tinct. Capsicum ... ... | 1½ minims. |
| Tinct. Can. Ind. ... ... | 46 minims. | Hydrocyanic Acid (dilute) | 9 ,, |
| Chloroform ... ... ... | 46 ,, | Glycerine ... ... .. | 75 ,, |
| Oil Peppermint ... ... | 1½ ,, | Alcohol ... ... q.s. ad | 1 fl. oz. |

The adult dose is 15 minims.

It will be observed that the fluid is of simpler composition than that of the so-called Chlorodyne, and that glycerine is used as the solvent. There is no sediment. It should be a uniform and trustworthy preparation.

---

**Tincture Strophanthus. Tabloids: Magnesium Sulphite; Opium Tinct.; Pure Tar and Codeine; Improved Gregory Powder. Suppositories: Glycerine; Hazeline; Zyminised (Peptonised).**—BURROUGHS, WELLCOME & CO., London.—The names of most of these preparations sufficiently indicate their composition and uses. **Sulphite of Magnesium** as a local bactericide in the treatment of follicular tonsillitis, aphthæ, and sarcinæ, should be of utility, as it is easily decomposed on contact with the mucous membrane into sulphurous acid and oxide of magnesium. The tabloids are flavoured with peppermint, and sweetened, so that no objection need be raised to them even by children. The **Gregory Powder Tabloids** contain a proportion of sodium in place of magnesium carbonate. They appear to be an improvement on the old Gregory powder (pulv. rhei co.), and have the advantage of the tabloid form in being less nauseous than the powder which has nauseated so many generations of sick children.

---

**Liq. Cinchonæ Hydrobrom.; Syr. Cinchon. Hydrobrom.; Syr. Cinchonæ et Ferri Hydrobrom.; Vibrona (Cinchona Wine).**—FLETCHER, FLETCHER & CO., London.—Fletcher's **Syrups** are too well known to need description, and their names are a sufficient index to their therapeutic properties. The combination of cinchona and iron is an excellent preparation. **Cinchona Wine** has not been so much appreciated in this country as on the Continent. This preparation from the red cinchona bark is not unlikely to become quite a fashionable medicine.

---

**Lactopeptine Tablets.**—JOHN M. RICHARDS, London.—Each tablet contains five grains of the powder which has withstood the competition of twenty years. We rather prefer the powder form, as the tablet does not mix so readily with the food and will take a much longer time to dissolve. The powder mixes so readily with water, and is so free from unpleasantness of taste, that many patients will prefer it to the tablet in spite of the greater convenience of the latter for the regulation of the dose.

---

**Vimbos.**—SCOTTISH FLUID BEEF CO. LIMITED, Edinburgh.—This is a semi-fluid paste, containing nitrogenous organic matter amounting to nearly 51 per cent., with salines (including phosphates) amounting to 24 per cent., and traces of fatty matter with only 25 per cent. of water. When suspended in water and boiled it gives a considerable precipitate of albumen. Its odour and taste are similar to those of other beef extracts, but its composition shows it to contain nutritious matter which must give it an active force-producing value over and above the stimulating properties of beef extracts in general. It is put up in bottles and earthenware jars, and is a convenient and efficient storage of nutriment for those who require easily assimilated and nutritious food.

---

**Dr. Tibbles' Vi-Cocoa.**—THE VI-COCOA COMPANY, London.—This cocoa is prepared from hops, malt, kola, and cocoa. It is more than a beverage, and must be ranked as a food of a restorative and stimulating kind. It is rather more bitter to the taste than other preparations of cocoa, but it must be distinctly more stimulating and sustaining, especially for those who have to do much exhausting brain-work.

**Izal.**—Newton, Chambers & Co. Limited, Sheffield.—Izal is a non-poisonous disinfectant which is highly spoken of by Dr. Klein, and for surgical purposes it is praised by Mr. Bruce Clarke. It is a powerful and absolutely non-irritating germicide, which may be used internally if required. It is now coming largely into use.

---

**Absorbent Lint; Absorbent Wool; Rubber Plaster (Calico and Holland); Holland Strapping; Belladonna Plaster; Open Wove Bandages (White and Grey).**—Leslies Limited, London.—Specimens of all the above have been sent us, and we can recommend them as the very best of their kind. They are too well known by all surgeons to need any description.

---

**Barff Boro-Glyceride.**—The Kreochyle Co., London.—This antiseptic food-preserver is now well known. It has also many surgical uses. The specimen sent to us is in a handy form of tube containing one ounce.

---

**Sedative Snuff; Antiseptic Handkerchiefs; Massage Unguent; Iodo-Glycerol Liniment; Ung. Hamamelidis Co.; Aseptic Hypodermic Solution of Morph. Hydroch.**—Arthur & Co., London.—The **Snuff** is the well known combination of bismuth and morphia (gr. ij. in ℥j.); it is a dry smooth powder with a pleasant aroma, which cannot fail to give satisfactory results when used with a ball insufflator in cases of nasal catarrh. The **Antiseptic Handkerchiefs** are made of tough absorbent Japanese paper, rendered aseptic and perfumed with eucalyptus, pinol, and lavender. They are decidedly an improvement on the ordinary handkerchiefs, which when dried or in the process of washing are likely to give rise to the dissemination of bacilli. Nothing is more needful in the management of cases of phthisis than the destruction of the discharged virus, and the use of cheap destructible handkerchiefs like these, which cost less than washing, enables the patient to accomplish the end in view. It is obvious that they will not disinfect the discharges, and must themselves be destroyed before the accumulated secretion can be converted into dust. The portion of the handkerchief used should be at once burnt, and not allowed previously to become dry. The **Massage Unguent** is a softened and deodorised oil of theobroma, the two faults in the ordinary oil having been removed. The **Iodo-Glycerol Liniment** is a non-irritating solution of iodine which is much more likely than the ordinary tinctures to be absorbed through the skin. The **Ung. Hamamelidis Co.** has a lanoline basis, with antiseptics and anodynes: it is put up in collapsible tubes, and should be very useful in the treatment of hemorrhoids. The **Aseptic Solution of Morphia** contains gr. $\frac{1}{20}$ in each minim: it is intended for hypodermic injection.

**The "Limpet" Air-tight Cover.**—Day & Co., Weston-super-Mare.—This is a very ingenious little device by means of which, with the most simple application, a cup or glass can be provided with a thoroughly efficient cover. It consists of a disc of rubber, tightly stretched in a circle of metal, and is applied by slight pressure with the fingers in the centre. It needs only to be known to come into general use. The cover is made in four sizes, which vary from 3⅝ to 7 inches in diameter. One or more of them should be in every house.

